# Effects of organic macro and trace minerals in fast and slower growing broiler breeders’ diet on offspring growth performance and tibia characteristics

**DOI:** 10.1016/j.psj.2021.101647

**Published:** 2021-12-09

**Authors:** B.C. Güz, I.C. de Jong, U.E. Bol, B. Kemp, M. van Krimpen, R. Molenaar, H. van den Brand

**Affiliations:** ⁎ Adaptation Physiology Group, Wageningen University and Research, Wageningen 6700 AH, the Netherlands; † Wageningen Livestock Research, Wageningen University and Research, Wageningen 6700 AH, Gelderland, the Netherlands

**Keywords:** organic, minerals, tibia, broiler breeder, broiler chicken

## Abstract

This study was designed to evaluate effects of source of macro and trace minerals (inorganic vs. organic) in fast and slower growing broiler breeders’ diets on egg and hatchling mineral content and on offspring tibia morphological, biophysical, and mechanical characteristics. After 10 wk feeding the breeders (at 30 wk of age), eggs were collected and incubated. Eggs and hatchlings were analysed on mineral content. Male chickens were assigned to 32 pens with 12 chickens per pen. At approximately 1,700 and 2,600 gram BW, three chickens per pen were slaughtered. Tibia characteristics were determined. Organic minerals in the broiler breeder diet resulted in higher Fe and Se concentration in the egg and in higher Se concentration in the hatchling. Despite effects of mineral source on mineral concentration in the eggs and hatchlings were limited, organic minerals in the slower-growing broiler breeder diet resulted in higher offspring BW (d 42, Δ = 115 g; *P* = 0.03) and advanced tibia development (higher thickness (∆ = 0.38 cm; *P* < 0.001), osseous volume (∆ = 5.1 cm^3^; *P* = 0.01), and mineral density (Δ = 0.13 g/cm^3^; *P* = 0.03) at 2,600 g BW), but this was not observed in fast-growing chickens. This suggests that 1) the difference in feed intake of the breeders between strains might affect offspring performance, which might indicate that current slower-growing breeder diets might be suboptimal in minerals or that transgenerational mineral availability in slower growing chickens appears to be more effective on bone development, which might be related to time available for bone development. 2) transgenerational mineral availability in offspring appears to play a role via other mechanisms than via absolute mineral concentrations.

## INTRODUCTION

In modern broiler chickens, the prevalence of suboptimal leg health is considerable (Bessei, 2006; Knowles et al., 2008; Gocsik et al., 2017). A potential reason is an imbalance between a high growth rate and insufficiently developed leg bones (Williams et al., 2000; Sherlock et al., 2010; Gonzalez-Ceron et al., 2015). Suboptimal leg health negatively affects broiler chickens’ locomotion (Bessei, 2006; Gocsik et al., 2017), welfare (Bradshaw et al., 2002; Mench, 2004) and slaughter revenues as a consequence of higher mortality and increased rejections at slaughter plants (Sullivian, 1994; Kestin et al., 1999; Mench, 2004; Grandin, 2010).

Fast-growing broiler chickens reach an average slaughter weight of 2,500 to 3,000 g in approximately 38 to 42 d, while slower growing broiler chickens reach the same weight in approximately 48 to 54 d (Quentin et al., 2004; Grashorn, 2006; Kokoszyński et al., 2017). Genetic selection for fast growth in broiler chickens has resulted in high feed efficiency and shorter rearing period, but also in more porous and less mineralized leg bones than slower growing broiler chickens (Lilburn, 1994; Dixon, 2020; Rayner et al., 2020). Consequently, fast-growing broilers have more leg and locomotion problems than slower growing broilers (Sullivian, 1994; Thorp and Waddington, 1997; Williams et al., 2000; Kokoszyński et al., 2017; Torres and Korver, 2018), expressed a higher risk of lameness and bone breakage (Williams et al., 2004; Stojcic and Bessei, 2009; Shim et al., 2012a).

One of the most important factors for bone development is the macro (Ca and P) and trace (Fe, Cu, Mn, Zn, Se) mineral availability (Bao et al., 2007; Yenice et al., 2015). Macro minerals are main components of the bone matrix (Rath et al., 1999; Blake and Fogelman, 2002), whereas both macro (Rosol and Capen, 1997; McDevitt et al., 2006) and trace (Richards, 1997; Angel, 2007; Dibner et al., 2007) minerals are essential bone development related hormones and enzymes. Mineral availability is not only determined by the amount of minerals provided in the diet, but also by the source or origin of the minerals. Minerals used in poultry diets mostly consist of an inorganic form (Van der Klis et al., 2002; Vieira, 2008). However, minerals can also be processed and bound to for example, an amino acid or a protein (Khatun et al., 2019; Wang et al., 2019) and are then called organic minerals. Organic macro and trace minerals in broiler's diet (Huang et al., 2009; Zhao et al., 2010) and organic trace minerals in broiler breeders’ diet (Bao and Choct, 2009; Swiatkiewicz et al., 2014; Torres and Korver, 2018) have been shown to have a higher bioavailability than inorganic minerals, due to a higher chemical stability and consequently a better intestinal absorption (Wedeking et al., 1991; Bao and Choct, 2009; Wang et al., 2019).

Pre-packaged minerals in the egg are important for embryonic bone development and later life bone health (Kidd et al., 1992; Dibner et al., 2007). Newly hatched chickens with a strong and well-formed skeletal frame might be able to move better and have lower prevalence of leg problems in later life (Torres and Korver, 2018). Organic trace minerals in the breeders’ diet have already been shown to result in better embryonic bone mineralization than inorganic trace minerals (Torres and Korver, 2018). However, these carry-over effects from breeders’ diet to the offspring embryo are only studied for trace minerals and not for macro minerals. Additionally, effects of mineral source in the breeder diet on offspring bone development and leg health at slaughter age are also hardly studied. Moreover, almost all studies on transgenerational effects of minerals in broiler chickens were conducted in fast-growing broiler breeders and not in slower growing broiler breeders.

The aim of this study was therefore to investigate effects of mineral source (inorganic vs. organic) in fast and slower-growing broiler breeders’ diet on egg and hatchling mineral content and on offspring tibia morphological, biophysical, and mechanical characteristics.

## MATERIALS AND METHODS

### Experimental Design

The experiment was setup as a 2 × 2 factorial arrangement with 2 broiler breeder strains (fast and slower growing) and two macro and trace mineral sources in the broiler breeder diets (organic and inorganic). Effects of the broiler breeder diet were investigated in the eggs and offspring (hatchlings and later life). During offspring rearing, no treatments were applied. The experiment was conducted at the experimental facility of Wageningen University and Research (Wageningen, the Netherlands). All procedures in this study were approved by the Central Commission on Animal Experiments (The Hague, the Netherlands; approval number: 2016.D-0138.005).

### Breeder Feeding Phase and Experimental Diets

At an age of 20 wk, 132 fast-growing Ross 308 breeders (120 females and 12 males) and 132 slower growing Hubbard JA57 breeders (120 females and 12 males) were obtained from 2 commercial rearing farms. Breeders were allocated to 8 pens (4 pens per strain with 30 females and 3 males per pen) and provided with a pre-layer diet (different for fast and slower growing breeders, following the recommendations of each strain; see Table 1). After 5 wk of adaptation, they were provided with one of 2 different layer diets (2 pens per strain per diet), containing either inorganic or organic macro (Ca, P) and trace (Fe, Cu, Mn, Zn, Se) minerals for a period of 10 wk (25 to 34 wk of age). Composition of these layer diets differed between fast and slower growing broiler breeders (Table 1), following the recommendations of each strain. Consequently, 4 treatment groups were used: 1) Fast-growing broiler breeders fed with inorganic macro and trace minerals (**IF**); 2) Fast-growing broiler breeders fed with organic macro and trace minerals (**OF**); 3) Slower growing broiler breeders fed with inorganic macro and trace minerals (**IS**); 4) Slower growing broiler breeders fed with organic macro and trace minerals (**OS**). Inorganic macro minerals (Ca and P) were provided by limestone and monocalcium phosphate. In the organic diets, Ca and P were largely (see Table 1) replaced by Calfos (Sonac Vuren B.V., Vuren, the Netherlands), a hydroxyapatite form of Ca and P, originating from processed porcine bones. Inorganic trace minerals (Fe, Cu, Mn, Zn, Se) were completely replaced by an organic sourced trace mineral premix (Optimin, Trouw Nutrition, Tilburg, the Netherlands). All other raw materials remained the same in both diets, although inclusion levels were different. Breeder diets were produced by ForFarmers N.V. (Heijen, the Netherlands) and analyzed for Ca, P, Fe, Cu, Mn, Zn, and Se (NutriControl, Veghel, the Netherlands). Diet compositions and calculated and analyzed nutrient values are shown in Table 1.

**Table 1:** Composition (%), calculated and analyzed nutrients of the experimental broiler breeder diets (g/kg, as-fed basis).

Ingredients		Slower growing strain			Fast-growing strain		
		Pre-lay	Inorganic	Organic	Pre-lay	Inorganic	Organic
Wheat	g/kg	311.0	200.0	210.7	180.0	180.0	180.0
Corn	g/kg	300.0	367.7	355.9	397.6	423.6	429.1
Barley	g/kg	50.0	44.6	50.0	50.0	45.5	50.0
Rapeseed meal	g/kg	30.0	50.0	50.0	3.00	50.0	50.0
Soybean meal HiPro	g/kg	49.1	91.7	88.3	30.0	55.0	50.9
Sunflowerseed meal HP	g/kg	150.0	125.0	125.0	132.5	125.0	125.0
Wheat bran	g/kg	23.7	-	-	97.4	0.50	-
Oat hulls	g/kg	30.0	10.0	10.0	30.0	10.0	10.0
Soy oil	g/kg	-	14.4	14.7	-	12.5	12.4
Crude palm oil	g/kg	8.0	3.1	2.8	0.80	5.0	5.1
Sodiumbicarbonate	g/kg	3.2	3.0	2.8	0.30	3.6	3.4
Limestone	g/kg	26.6	71.5	-	24.2	66.3	-
Oystershell	g/kg	-	-	68.5	-	-	62.9
Monocalciumphosphate	g/kg	3.5	3.7	-	3.5	4.1	
Calfos^1^	g/kg	-	-	5.9	-	-	6.6
Salt	g/kg	1.7	1.8	1.8	2.1	1.7	1.6
L-Lysine HCl	g/kg	1.9	1.1	1.1	0.7	0.8	0.9
DL-methionine	g/kg	0.4	1.5	1.5	0.1	0.7	0.7
L-threonine	g/kg					0.3	0.3
Xylanase 6,25%	g/kg	1.0	1.0	1.0	1.0	1.0	1.0
Premix Organic^2^	g/kg	-	-	10.0	-	-	10.0
Premix Inorganic^3^	g/kg	10.0	10.0	-	10.0	10.0	-
Total	g/kg	1000	1000	1000	1000	1000	1000
Calculated nutrients							
AMEn layer	kcal/kg	2750	2750	2750	2750	2800	2800
Crude protein	g/kg	160	165	165	145	148	148
Crude fat	g/kg	37	46	46	40	47	48
Crude fiber	g/kg	59	48	49	61	48	48
Crude ash	g/kg	70	114	113	68	107	106
Starch Brunt	g/kg	409	381	382	405	406	411
Calcium	g/kg	15.0	32.0	32.0	14.0	30.0	30.0
Phosphorus	g/kg	5.2	5.0	5.0	5.4	5.0	4.9
retainable P	g/kg	3.2	3.2	3.2	3.2	3.2	3.2
Sodium	g/kg	1.6	1.6	1.6	1.7	1.7	1.7
Chloride	g/kg	2.1	2.0	2.0	2.2	1.9	1.9
Dig.lysine	g/kg	6.5	6.7	6.7	5.0	5.6	5.6
Dig.methionine	g/kg	3.0	4.1	4.1	2.5	3.1	3.1
Dig.met+cys	g/kg	5.6	6.7	6.7	4.9	5.5	5.5
Dig.threonine	g/kg	4.5	4.9	4.9	4.1	4.6	4.6
Dig.tryptophan	g/kg	1.65	1.71	1.70	1.51	1.50	1.48
Dig.isoleucine	g/kg	5.1	5.5	5.5	4.6	4.8	4.8
Dig.valine	g/kg	6.4	6.7	6.6	5.8	6.0	6.0
Analyzed nutrients							
Ca	g/kg	-	30.1	30.2	-	30.3	27.4
P	g/kg	-	5.74	4.98	-	5.46	4.99
Fe	mg/kg	-	323	312	-	275	320
Cu	mg/kg	-	18	19	-	15	19
Mn	mg/kg	-	85	99	-	95	86
Zn	mg/kg	-	82	79	-	77	75
Se	mg/kg	-	0.35	0.38	-	0.35	0.39

1 Composition of Calfos provided per kg of product: 100 g crude protein, 300 g calcium, 130 g phosphorus (113 g digestible phosphorus), 50 g moisture.

2 Composition of organic premix provided per kg of diet: 12,000 IU vitamin A (source of vitamin A), 2,400 IU vitamin D3, 30 IU vitamin E (source of vitamin E), 1.5 mg vitamin K3, 2 mg vitamin B1, 7.5 mg vitamin B2, 10 mg d-pantothenic acid, 35 mg niacin amide, 200 μg biotin, 20 μg vitamin B12, 1 mg folic acid, 3.5 mg vitamin B6, 461 mg choline chloride, 80 mg Fe (as Fe proteinate), 12 mg Cu (as Cu proteinate), 60 mg Zn (as Zn proteinate), 85 mg Mn (as Mn proteinate), 0.4 mg Co (as CoSO_4_-7H_2_O), 0.8 mg I (as KI), 0.1 mg Se (as Se selenite) and 50 mg antioxidant.

3 Composition of inorganic premix provided per kg of diet: 12,000 IU vitamin A (source of vitamin A), 2,400 IU vitamin D3, 30 IU vitamin E (source of vitamin E), 1.5 mg vitamin K3, 2 mg vitamin B1, 7.5 mg vitamin B2, 10 mg d-pantothenic acid, 35 mg niacin amide, 200 μg biotin, 20 μg vitamin B12, 1 mg folic acid, 3,5 mg vitamin B6, 461 mg choline chloride, 80 mg Fe (as FeSO4-H2O), 12 mg Cu (as CuSO_4_-5H_2_O), 60 mg Zn (as ZnSO_4_-H_2_O), 85 mg Mn (as MnO), 0.4 mg Co (as CoSO_4_-7H_2_O), 0.8 mg I (as KI), 0.1 mg Se (as Na_2_SeO_3_-5H_2_O), and 50 mg antioxidant.

### Egg Collection, Storage, and Mineral Analysis

In wk 34 of breeders’ age, first grade eggs were collected for 5 d, transferred to a storage room and stored at 15 to 16°C and a relative humidity of 65 to 70%. In the same week, 5 eggs per treatment (1 egg per day for 5 d) were collected and analysed for macro (Ca and P) and trace (Fe, Cu, Mn, Zn, Se) minerals by a commercial laboratory (NutriControl), using inductively coupled plasma – optical emission spectrometry (**ICP-OES**).

### Incubation and Hatching Phase

After a storage duration of 4 to 8 d, 60 eggs per treatment per collection day (300 eggs per treatment, 1,200 eggs in total) were randomly distributed over 4 incubators; 2 large incubators with 440 eggs each (8 trays of 55 eggs per tray) and 2 small incubators with 160 eggs each (4 trays of 40 eggs per tray). Each incubator contained eggs from all 4 treatments and each tray contained eggs of one treatment. Per incubator, 4 or 5 eggshell temperature (**EST**) sensors (Pt-100, Sensor Data BV, Rijswijk, the Netherlands) were attached to 4 or 5 individual eggs. All sensors were placed at the equator of the chosen eggs, using heat conducting paste (Dow Corning 340 Heat Sink Compound, Dow Corning GmbH, Wiesbaden, Germany) and a small piece of tape (2 × 2 cm). The incubator temperature was continuously adjusted based on the median temperature of the EST sensors to maintain an EST of 37.8°C throughout incubation. Relative humidity of incubators was maintained between 50 and 65% throughout incubation. Eggs were turned every 30 min at an angle of 90^o^ and not exposed to light during incubation. At embryonic d 8 (**E8**), all eggs were candled and infertile eggs were removed. At E18, all eggs were candled again and eggs containing a vital embryo were transferred from trays to hatching baskets, which were placed back in the same incubator.

After all chickens had hatched, they were taken from the hatching baskets (d E21.5). Chicken quality parameters (red hock, red beak and navel score) of all hatched chickens (both males and females, n = 1,032) were assessed. Red hock and red beak were scored as 0 or 1 (absent or present). Navel score was assessed as 1 (good), 2 (moderate), or 3 (poor) as described by (Molenaar et al., 2010). After chicken quality assessment, all chickens were feather-sexed. Male chickens were individually weighed and numbered by using a neck-label, vaccinated against infectious bronchitis (eye drop; MSD Animal Health, Boxmeer, the Netherlands) and transported to one of 2 adjacent rearing rooms at the same location.

After hatch, 5 randomly chosen male chickens per treatment were killed by cervical dislocation and residual yolks were removed. Yolk-free bodies and residual yolks were analyzed separately for macro (Ca and P) and trace (Fe, Cu, Mn, Zn, Se) minerals by a commercial laboratory (NutriControl), using ICP-OES.

### Broiler Rearing Phase

Upon arrival at the rearing rooms, male chickens per broiler breeder strain and diet were randomly assigned to 32 pens (16 pens per room) within 8 blocks of 4 pens (8 replicates per treatment), 12 chickens per pen. Pens (1.25 × 2.00 m) were covered with 4 to 6 cm wood shavings. Temperature was maintained at 32°C until d 3 of age and thereafter gradually reduced to 24°C at d 21 and to 20°C from d 28 onward. A continuous light program from arrival to d 3 of age and a 16L:8D light program from d 4 to 49 was applied. Chickens were raised from arrival to d 42 (fast-growing) or d 49 (slower growing) with ad libitum access to a similar commercially available diet (ForFarmers, Lochem, the Netherlands). A 3-phase feeding program was applied; a starter diet (MEbroiler = 2925 kcal/kg, CP = 203 g/kg, dLys = 11.1 g/kg) was provided from d 0 to 14, a grower diet (MEbroiler = 2975 kcal/kg, CP = 173 g/kg, dLys = 9.1 g/kg) from y 14 to 35 and a finisher diet (MEbroiler = 3,025 kcal/kg, CP = 172 g/kg, dLys = 8.6 g/kg) from d 35 to 42 (fast-growing) or 49 (slower growing). Water was available ad libitum throughout the rearing phase via drinking nipples. At d 11, chickens were vaccinated against Newcastle disease (Clone 30; eye drop, MSD Animal Health).

### Data Collection, Sampling, and Measurements

All chickens were individually weighed on d 0, 10, 14, 21, 28, 35, 42, and 49 of age. Feed intake (**FI**) was measured per pen for the starter, grower, and finisher period and over the whole rearing period. Feed conversion ratios (**FCR**) were calculated for the same periods, taking mortality into account. Mortality was recorded per pen per day and dead chickens were weighed.

Home pen behaviour was scored on d 16, 23, 30, 37, and 44 with one morning and one afternoon session, using the scan sampling technique (De Jong and Gunnink, 2019). During 3 to 4 min per session per pen per day, the number of chickens performing the following activities was scored: eating, drinking, walking, standing, resting, foraging, sitting, dust bathing, or perching.

At a body weight (**BW**) of approximately 1,700 gram (d 29 and 38 of age for fast and slower growing chickens, respectively) and at a BW of approximately 2,600 gram (d 38 and 49 of age for fast and slower growing chickens, respectively) three chickens per pen were selected for slaughter. Chickens with a BW close to 1,700 or 2,600 g were selected. Chickens were stunned by electrocution, cut, and bled. Varus Valgus (**VV;** each angulation was scored as present, 1; or no angulation, 0) was scored on both legs by visual appraisal. The left leg of all chickens was assessed by a veterinarian on tibia dyschondroplasia (**TD**), bacterial chondronecrosis with osteomyelitis (**BCO**), epiphyseal plate abnormalities (**EPA**) and epiphysiolysis (**EPI**). These abnormalities were scored in the range of 0 (no abnormalities), 1 (minor abnormality), or 2 (severe abnormality), but analyzed as 0 (no abnormality) or 1 (abnormality), because no score 2 was observed.

The tibia of the right leg was obtained from each chicken and frozen at −20°C. After thawing, tibia of 2 chickens per pen was selected for further analyses. Tibia weight was determined. Tibia proximal length, lateral cortex thickness, femoral, and metatarsal side proximal head thickness, osseous volume, pore volume, total volume (osseous volume + pore volume), volume fraction (osseous volume / total volume), mineral content, and mineral density were analyzed on each tibia, using a GE Phoenix 3D X-ray microfocus CT scanner (General Electric Company, Boston, MA) (Bouxsein et al., 2010; Güz et al., 2019, 2021). Robusticity index was calculated, using the following formula (Riesenfeld, 1972):

Robusticityindex(cm/g)=boneproximallength(cm)/boneweight(g).

The same tibias were then subjected to a three-point bending test (Jungmann et al., 2011), using an Instron electromechanical universal testing machine (Instron, Norwood, MA). Ultimate strength (maximal load at breaking point); yield strength (reached yield load the angulation point on slope); tibia stiffness (the slope of the selected linear part of the curve), and energy to fracture (the area under the curve) were measured (Güz et al., 2019, 2021). Elastic modulus (GPa), which is the amount of strain caused by stress (Novitskaya et al., 2011), was calculated using the following formula (Turner and Burr, 1993):

$$E=\frac{N\;S^{3}}{4\delta \;TL^{3}}$$

where *E* is the elastic modulus (GPa), *N* is the maximal load (N), S is the span between bending fixtures (mm), T is the tibia thickness (mm), L is the tibia length (mm) and δ is the maximum deflection (mm) at the midpoint of the bone.

### Statistical Analysis

All statistical analyses were performed in SAS (Version 9.4, 2013, SAS Institute Inc., Cary, NC).

Hatch data (red hock, red beak, and navel score) was subjected to generalized linear mixed model analysis, using PROC GLIMMIX with model 1:

(1) Y=μ+Mineral+Strain+Interaction+ε,

where Y = the dependent variable, μ is the overall mean, Mineral = mineral source in the broiler breeder diet (organic or inorganic), Strain = broiler breeder strain (fast-growing Ross 308 or slower-growing Hubbard JA757), Interaction = interaction between mineral source and strain, Ɛ = residual error. Incubator was added to the model as a random effect.

Red hock and red beak were analysed at binary level (present or not) with a logit link; navel score was analyzed at multinomial level (good, moderate, or poor) with a cumlogit link. Hatchling was used as the experimental unit.

All growth performance data from d 0 to 42 (BW, FI, FCR, mortality) was subjected to general linear mixed model analysis, using PROC MIXED with model 1. Pen was used as the experimental unit. Block was used as a random factor.

From d 42 onward, only chickens from the slower growing strain were present. Consequently, all growth performance data (BW, FI, FCR, mortality) was subjected to general linear mixed model analysis, using PROC MIXED with model 2. Pen was used as the experimental unit. The statistical model used was:

(2) Y=μ+Mineral+ε,

where Y = the dependent variable, μ is the overall mean, Mineral = mineral source in the broiler breeder diet (organic or inorganic), Ɛ = residual error. Block was used as a random factor.

Tibia morphological, biophysical, and mechanical characteristics, at 2 BW classes (1,700 and 2,600 g), were subjected to general linear mixed model analysis, using PROC MIXED with model 1, added with BW (actual BW of the individual chickens at slaughter age) as a covariate. Pen was used as the experimental unit. Block was used as a random factor. Chicken nested within pen was added as a random factor to use pen as the experimental unit in the analyses.

Home pen behaviour (eating, drinking, walking, standing, resting, sitting, dust bathing, and perching) was subjected to general linear mixed model analysis, using PROC MIXED with model 1. Pen was used as the experimental unit. Block was used as a random factor. Only main effects were analysed and presented, because preliminary analyses demonstrated a lack of interaction effects between mineral source and breeder strain.

Leg disorders (VV, TD, EPA, BCO, and EPI) were subjected to generalized linear mixed model analysis, at 2 BW classes (1,700 and 2,600 g), using PROC GLIMMIX with model 1. VV was scored as present or not (each angulation was scored as 0 (no angulation) or 1 (angulation present). TD was scored in the range of 0 (no abnormalities), 1 (minor abnormality), or 2 (severe abnormality), but analyzed as 0 (no abnormalities) or 1 (abnormalities present), because no score 2 was found. For all these analyses, a logit link was used. Pen was used as the experimental unit. EPA, BCO and EPI were not statistically analyzed, because there was only one BCO disorder in the OF group at 1,700 g BW class and no observations were recorded for EPA and EPI.

For continuous data, model assumptions were checked for both means and residuals. Non-normal distributed data were log-transformed before analyses. Results are provided as LSmeans ± SEM, unless indicated otherwise. When multiple comparisons were performed, the level of significance was corrected, using Bonferroni. Effects were considered to be significant at *P* ≤ 0.05.

## RESULTS

### Mineral Analysis

By incidence, eggs selected for mineral analyses differed in weight between mineral source in the breeder diet and did not represent the average egg weight of the treatments. Consequently, egg mineral composition is expressed as concentration. No interaction effects between mineral source and breeder strain were found in mineral concentrations of albumen + yolk, eggshell, residual yolk or yolk free body (Tables 2 and 3). Iron (∆ = 3.2 mg/kg, *P* = 0.008) and selenium (∆ = 0.073 mg/kg, *P* < 0.001) were higher in albumen + yolk of eggs originating from organic minerals fed broiler breeders compared to eggs of inorganic minerals fed broiler breeders, whereas the opposite was found for copper (∆ = 0.1 mg/kg, *P* = 0.05; Table 2). No strain effect was found for albumen + yolk mineral concentrations.

**Table 2:** Effects of broiler breeder strain (fast-growing Ross 308 or slower growing Hubbard JA 757), dietary mineral source (inorganic or organic) and their interaction on analyzed egg mineral concentrations (albumen + yolk and eggshell, separately) (n = 5 eggs per treatment, LSmeans ± SEM).

Material	EW^1^	Albumen and yolk								Eggshell		
Parameter		AYW^1^	Ca^2^	P^2^	Fe^2^	Cu^2^	Mn^2^	Zn^2^	Se^2^	ESW^1^	Ca^2^	P^2^
Mineral source												
Inorganic	50.30^b^	43.93^b^	0.058	0.205	22.8^b^	0.8^a^	0.3	15.9	0.306^b^	6.37	30.3	0.128
Organic	57.98^a^	51.37^a^	0.060	0.213	26.0^a^	0.7^b^	0.3	16.9	0.379^a^	6.61	29.4	0.119
SEM	1.16	1.04	0.001	0.003	0.7	0.1	0.1	0.4	0.012	0.18	0.4	0.005
Strain												
Fast	54.74	48.21	0.058	0.209	24.2	0.7	0.3	16.1	0.332	6.53	30.4	0.124
Slower	53.54	47.09	0.060	0.208	24.6	0.7	0.3	16.7	0.352	6.45	29.3	0.123
SEM	1.19	1.04	0.001	0.003	0.7	0.1	0.1	0.4	0.012	0.18	0.4	0.005
Mineral source × strain												
Inorganic fast	52.16	45.66	0.057	0.207	22.2	0.8	0.2	15.3	0.292	6.50	30.6	0.129
Organic fast	57.32	50.76	0.059	0.212	26.2	0.7	0.3	16.8	0.373	6.56	30.2	0.118
Inorganic slower	48.44	42.20	0.059	0.203	23.3	0.7	0.3	16.5	0.320	6.24	29.9	0.127
Organic slower	58.64	51.98	0.062	0.214	25.8	0.7	0.3	16.9	0.385	6.66	28.6	0.120
SEM	1.41	1.48	0.002	0.005	1.1	0.1	0.1	0.5	0.017	0.26	0.6	0.007
*P*-values												
Mineral source	0.03	0.001	0.22	0.09	0.008	0.05	0.50	0.08	<0.001	0.36	0.20	0.19
Strain	0.62	0.46	0.26	0.82	0.76	0.68	0.82	0.26	0.26	0.76	0.09	0.95
Mineral source × strain	0.11	0.14	0.79	0.55	0.51	0.41	0.13	0.32	0.67	0.49	0.47	0.76

a-b Values within a column and factor lacking a common superscript differ (*P* ≤ 0.05).

1 EW: Total egg weight (Albumen and yolk weight + eggshell weight) (g); AYW: Albumen and yolk weight (g); ESW: Eggshell weight (g).

2 The unit for Ca and P is g/100 g; the unit for Fe, Cu, Mn, Zn, and Se is mg/kg.

**Table 3:** Effects of broiler breeder strain (fast-growing Ross 308 or slower growing Hubbard JA 757), dietary mineral source (inorganic or organic) and their interaction on analyzed mineral contents of residual yolk and yolk free body of hatchling (n = 5 hatchlings per treatment, LSmeans ± SEM).

Material	HW^1^	Residual yolk								Yolk free body							
Parameter		RYW^1^	Ca^2^	P^2^	Fe^2^	Cu^2^	Mn^2^	Zn^2^	Se^2^	YFBW^1^	Ca^2^	P^2^	Fe^2^	Cu^2^	Mn^2^	Zn^2^	Se^2^
Mineral source																	
Inorganic	44.10	3.57	1.36	0.19	16.9	0.8	1.0	23.6	1.08^b^	40.52	0.28	0.27	29.7	0.9	0.2	18.8	0.32^b^
Organic	43.76	3.61	1.50	0.18	17.4	0.8	1.2	21.8	1.19^a^	40.15	0.29	0.27	30.6	0.9	0.3	17.6	0.36^a^
SEM	0.96	0.18	0.059	0.009	1.4	0.1	0.1	1.7	0.04	0.64	0.012	0.007	0.4	0.1	0.1	0.6	0.01
Strain																	
Fast	46.51^a^	3.89^a^	1.10^b^	0.20^a^	21.0^a^	0.7^b^	0.9^b^	22.9	1.23^a^	42.61^a^	0.29	0.28	32.3^a^	0.9	0.3	19.0	0.36^a^
Slower	41.35^b^	3.29^b^	1.75^a^	0.16^b^	13.3^b^	0.9^a^	1.3^a^	22.5	1.04^b^	38.06^b^	0.28	0.27	27.9^b^	0.9	0.3	17.4	0.32^b^
SEM	0.93	0.18	0.059	0.009	1.4	0.1	0.1	1.7	0.04	0.64	0.012	0.007	0.4	0.1	0.1	0.6	0.01
Mineral source × strain																	
Inorganic fast	47.08	4.00	1.03	0.21	19.8	0.7	0.9	23.1	1.17	43.08	0.29	0.28	31.8	0.9	0.3	20.3	0.32
Organic fast	45.92	3.78	1.18	0.20	22.1	0.6	1.0	22.7	1.29	42.14	0.29	0.27	32.9	0.9	0.3	17.8	0.40
Inorganic slower	41.11	3.14	1.68	0.17	13.9	0.9	1.2	24.1	0.98	37.96	0.27	0.26	27.6	0.9	0.2	17.4	0.31
Organic slower	41.59	3.44	1.82	0.16	12.6	0.9	1.5	20.8	1.10	38.16	0.29	0.27	28.3	0.9	0.3	17.4	0.33
SEM	1.15	0.25	0.083	0.013	2.0	0.1	0.1	2.4	0.05	0.90	0.017	0.009	0.6	0.1	0.1	0.8	0.02
*P*-values																	
Mineral source	0.47	0.88	0.11	0.47	0.82	0.85	0.08	0.45	0.05	0.69	0.60	0.83	0.12	0.87	0.33	0.14	0.009
Strain	0.03	0.04	<0.001	0.009	0.002	0.05	0.003	0.87	0.003	0.001	0.56	0.39	<0.001	0.62	1.0	0.07	0.03
Mineral source × strain	0.18	0.33	0.94	0.66	0.39	0.56	0.31	0.57	0.97	0.54	0.59	0.35	0.77	0.62	0.51	0.14	0.17

a-b Values within a column and factor lacking a common superscript differ (*P* ≤ 0.05).

1 HW: Hatchling weight (residual yolk weight + yolk free body weight) (g); RYW: Residual yolk weight (g); YFBW: Yolk free body weight (g).

2 The unit for Ca and P is g/100 g; the unit for Fe, Cu, Mn, Zn, and Se is mg/kg.

Selenium concentration was higher (∆ = 0.11 mg/kg, *P* = 0.05) in residual yolk of hatchlings originating from organic minerals fed broiler breeders compared to inorganic minerals fed broiler breeders (Table 3). In the residual yolk, calcium (∆ = 0.65 g/kg, *P* < 0.001), copper (∆ = 0.2 mg/kg, *P* = 0.05), and manganese (∆ = 0.4 mg/kg, *P* = 0.003) concentrations were lower in fast-growing chickens compared to slower growing chickens, whereas the opposite was found for phosphorus (∆ = 0.04 g/kg, *P* = 0.009), iron (∆ = 7.7 mg/kg, *P* = 0.002), and selenium (∆ = 0.19 mg/kg, *P* = 0.003) concentrations.

Selenium concentration was higher (∆ = 0.04 mg/kg, *P* = 0.009) in yolk-free bodies of hatchlings originating from organic minerals fed broiler breeders compared to inorganic minerals fed broiler breeders. Iron (∆ = 4.4 mg/kg, *P* < 0.001) and selenium concentration (∆ = 0.4 mg/kg, *P* = 0.03) were lower in yolk-free bodies of slower growing hatchlings compared to fast-growing hatchlings (Table 3).

### General Hatch Data

Hatchability of fertile eggs was on average 86%. Hatch characteristics (red hock, red beak, and navel score of all chickens) are shown in Table A1. No interaction effects between mineral source and breeder strain or mineral source effects were found for red hock, red beak, and navel score. Slower growing chickens had a higher incidence of red hocks (∆ = 3.73 %, *P* = 0.02) and red beaks (∆ = 3.06 %, *P* = 0.05) than fast-growing chickens. Navel score was not affected by breeder strain.

### Growth Performance

BW at d 10, 14, 21, 28, 35, and 42 of the broiler rearing phase showed a significant interaction between mineral source and breeder strain (Table 4). In fast-growing chickens, no effect of mineral source was found on BW at any of the weighing days, but slower growing chickens originating from organic minerals fed broiler breeders had a higher BW at d 10 (Δ = 20 g; *P* = 0.04), d 14 (Δ = 20 g; *P* = 0.04), d 21 (Δ = 39 g; *P* = 0.04), d 28 (Δ = 65 g; *P* = 0.04), d 35 (Δ = 78 g; *P* = 0.03), and d 42 (Δ = 115 g; *P* = 0.03) than the ones originating from inorganic mineral fed broiler breeders. Slower growing chickens originating from organic mineral fed broiler breeders had a higher BW at d 49 (∆ = 132 g, *P* = 0.004) than chickens originating from inorganic minerals fed broiler breeders. Chickens of the slower growing strain had a lower BW than fast-growing strain at d 0 (∆ = 3.4 g, *P* < 0.001).

**Table 4:** Effects of broiler breeder strain (fast-growing Ross 308 or slower growing Hubbard JA 757), dietary mineral source (inorganic or organic) and their interaction on BW (gram) of male broiler offspring at different ages (n = 8 pens per treatment, LSmeans ± SEM).

Parameter	D 0	D 10	D 14	D 21	D 28	D 35	D 42	D 49*
Mineral source								
Inorganic	40.9	215	376	727	1,321	2,005	2,561	-
Organic	40.5	224	385	744	1,356	2,033	2,604	-
SEM	0.2	4	4	8	10	14	22	-
Strain								
Fast	42.4^a^	248	440	853	1,620	2,439	3,115	-
Slower	39.0^b^	191	321	617	1,056	1,598	2,051	-
SEM	0.2	4	4	8	10	14	22	-
Mineral source × strain								
Inorganic fast	42.3	250^a^	441^a^	855^a^	1,618^a^	2451^a^	3,129^a^	-
Organic fast	42.4	246^a^	438^a^	851^a^	1,623^a^	2,,428^a^	3,100^a^	-
Inorganic slower	39.4	181^c^	311^c^	598^c^	1,024^c^	1,559^c^	1,993^c^	2619^b^
Organic slower	38.6	201^b^	331^b^	637^b^	1,089^b^	1,637^b^	2,108^b^	2751^a^
SEM	0.3	5	6	11	14	20	31	30
*P*-values								
Mineral source	0.17	0.12	0.12	0.08	0.03	0.20	0.18	0.004
Strain	<0.001	<0.001	<0.001	<0.001	<0.001	<0.001	<0.001	-
Mineral source × strain	0.08	0.04	0.04	0.04	0.05	0.03	0.03	-

a-c Values within a column and factor lacking a common superscript differ (*P* ≤ 0.05).

⁎ At d 49, only chickens of the slower growing strain were present.

Feed intake between d 0 and 14 showed a significant interaction between mineral source and breeder strain (Table 5), but this effect disappeared after correction for Bonferroni. No further interactions between mineral source and breeder strain were found for FI or FCR and neither mineral source effects were found. Slower growing chickens had a lower feed intake between d 14–35 (∆ = 906 g, *P* < 0.001), 35–42 (∆ = 253 g, *P* < 0.001), and 0–42 (∆ = 1270 g, *P* < 0.001) than fast-growing chickens. Slower growing chickens had a higher FCR than fast-growing chickens between da 0–14 (∆ = 0.10, *P* < 0.001), 14–35 (∆ = 0.16, *P* < 0.001), 35–42 (∆ = 0.30, *P* < 0.001) and 0–42 (∆ = 0.18, *P* < 0.001).

**Table 5:** Effects of broiler breeder strain (fast-growing Ross 308 or slower growing Hubbard JA 757), dietary mineral source (inorganic or organic) and their interaction on feed intake (gram per chicken) and feed conversion ratio (FI/BWG) of male broiler offspring in different phases of the rearing period (n = 8 pens per treatment, LSmeans ± SEM).

Parameter	FI D 0–14	FI d 14–35	FI d 35–42	FI d 0–42^1^	FI d 42–49^2^	FI d 0–49^2^	FCR d 0–14	FCR d 14–35	FCR d 35–42	FCR d 0–42^1^	FCR d 42–49^2^	FCR d 0–49^2^
Mineral source												
Inorganic	416	2,609	1,050	4,076	-	-	1.25	1.62	1.93	1.64	-	-
Organic	419	2,647	1,064	4,130	-	-	1.23	1.62	1.89	1.62	-	-
SEM	6	30	15	42	-	-	0.01	0.01	0.04	0.01	-	-
Strain												
Fast	473	3,081^b^	1,184^a^	4,738^a^	-	-	1.19^b^	1.54^b^	1.76^b^	1.54^b^	-	-
Slower	362	2,175^a^	931^b^	3,468^b^	-	-	1.29^a^	1.70^a^	2.06^a^	1.72^a^	-	-
SEM	6	30	15	42	-	-	0.01	0.01	0.04	0.01	-	-
Mineral source × strain												
Inorganic fast	479^a^	3,090	1,195	4,765	-	-	1.20	1.54	1.77	1.54	-	-
Organic fast	467^a^	3,072	1,172	4,712	-	-	1.18	1.55	1.75	1.54	-	-
Inorganic slower	353^b^	2,128	905	3,387	1,194	4,581	1.31	1.71	2.09	1.74	1.91	1.78
Organic slower	371^b^	2,221	956	3,548	1,244	4,793	1.28	1.70	2.03	1.72	1.93	1.77
SEM	7	40	22	59	45	88	0.02	0.02	0.05	0.02	0.04	0.02
*P*-values												
Mineral source	0.67	0.34	0.53	0.38	0.22	0.09	0.08	0.88	0.49	0.58	0.81	0.76
Strain	<0.001	<0.001	<0.001	<0.001	-	-	<0.001	<0.001	<0.001	<0.001	-	-
Mineral source × strain	0.02	0.17	0.11	0.09	-	-	0.78	0.84	0.67	0.76	-	-

a-b Values within a column and factor lacking a common superscript differ (*P* ≤ 0.05).

1 Only fast-growing chickens.

2 Only slower growing chickens.

A total of 12 (3.1%) dead chickens were recorded during the rearing period. No interaction between mineral source and breeder strain or main effects were found on mortality.

### Tibia Morphological Characteristics

At the 1,700 g BW class, interaction effects between mineral source and broiler breeder strain were found on all tibia morphological characteristics, except for tibia lateral cortex thickness (Table 6). The OS group had a higher tibia weight compared to the other treatment groups (∆ = 0.86 g on average; *P* = 0.006), which were similar. For proximal tibia length, tibia femoral side head thickness, tibia metatarsal side head thickness and tibia robusticity index, no effects of mineral source were found in the slower growing broilers. However, in fast-growing broilers, chickens originating from organic minerals fed broiler breeders had higher proximal tibia length (Δ = 0.88 cm; *P* = 0.02), tibia femoral side head thickness (Δ = 0.21 cm; *P* = 0.03), tibia metatarsal side head thickness (∆ = 0.23 cm; *P*=0.04), and tibia robusticity index (∆ = 0.07 cm/g; *P* < 0.001) than chickens originating from inorganic minerals fed broiler breeders. For the 1,700 g BW class, the lateral tibia cortex thickness was higher in slower growing chickens than in fast-growing chickens (Δ = 0.17 cm; *P* < 0.001).

**Table 6:** Effects of broiler breeder strain (fast-growing Ross 308 or slower growing Hubbard JA 757), dietary mineral source (inorganic or organic) and their interaction on tibia morphological characteristics of male broiler offspring in two BW classes (1,700 and 2,600 g) (2 chickens per pen, n = 8 pens per treatment; LSmeans ± SEM).

Parameter	Tibia weight (g)		Proximal tibia length (cm)		Lateral tibia cortex thickness (cm)		Femoral side proximal tibia head thickness (cm)		Metatarsal side proximal tibia head thickness (cm)		Tibia robusticity index (cm/g)	
BW class	1,700 g	2,600 g	1,700 g	2,600 g	1,700 g	2,600 g	1,700 g	2,600 g	1,700 g	2,600 g	1,700 g	2,600 g
Mineral source												
Inorganic	13.56	16.25	11.59	12.79	1.26	1.30	3.55	3.97	3.20	3.58	0.86	0.79
Organic	13.98	16.54	12.09	12.87	1.31	1.50	3.70	4.10	3.33	3.66	0.87	0.78
SEM	0.2	0.1	0.1	0.1	0.02	0.02	0.03	0.03	0.03	0.03	0.01	0.01
Strain												
Fast	13.56	15.57^b^	10.85	12.68^b^	1.20^b^	1.24	3.32	3.90	2.99	3.49	0.81	0.82^b^
Slower	13.89	17.22^a^	12.83	12.98^a^	1.37^a^	1.56	3.94	4.18	3.53	3.75	0.92	0.75^a^
SEM	0.2	0.1	0.1	0.1	0.02	0.02	0.03	0.03	0.03	0.03	0.01	0.01
Mineral source × strain												
Inorganic fast	13.52^b^	15.39	10.41^c^	12.67	1.19	1.23^c^	3.19^c^	3.89^c^	2.88^c^	3.48^c^	0.77^c^	0.82
Organic fast	13.41^b^	15.75	11.29^b^	12.69	1.22	1.25^c^	3.44^b^	3.89^c^	3.11^b^	3.49^c^	0.84^b^	0.81
Inorganic slower	13.60^b^	17.10	12.78^a^	12.90	1.34	1.37^b^	3.92^a^	4.05^b^	3.51^a^	3.68^b^	0.94^a^	0.76
Organic slower	14.37^a^	17.34	12.89^a^	13.06	1.41	1.75^a^	3.96^a^	4.31^a^	3.56^a^	3.83^a^	0.90^a^	0.75
SEM	0.2	0.2	0.1	0.1	0.03	0.03	0.04	0.04	0.04	0.03	0.01	0.01
*P*-values												
Mineral source	0.04	0.06	0.003	0.49	0.07	<0.001	0.003	0.007	0.003	0.006	0.28	0.36
Strain	0.002	<0.001	<0.001	0.02	<0.001	<0.001	<0.001	<0.001	<0.001	<0.001	<0.001	<0.001
Mineral source × strain	0.006	0.70	0.02	0.55	0.43	<0.001	0.03	0.009	0.04	0.02	<0.001	0.45

a-c Values within a column and factor lacking a common superscript differ (*P* ≤ 0.05).

At 2,600 g BW class, interaction effects between mineral source and broiler breeder strain were found on lateral tibia cortex thickness, femoral side proximal tibia head thickness, and tibia metatarsal side proximal tibia head thickness (Table 6). In fast-growing chickens, no effects of mineral source in the breeder diet were found, but slower growing chickens, originating from organic minerals fed broiler breeders had a higher tibia lateral cortex thickness (∆ = 0.38 cm; *P* < 0.001), tibia femoral side proximal head thickness (∆ = 0.26 cm; *P* = 0.009), and tibia metatarsal side proximal head thickness (∆ = 0.15 cm; *P* = 0.02) than chickens originating from inorganic minerals fed broiler breeders. Slower growing chickens showed a higher tibia weight (∆ = 1.65 g; *P* < 0.001), proximal tibia length (∆ = 0.30 cm; *P* = 0.02), and a lower tibia robusticity index (∆ = 0.07 cm/g; *P* < 0.001) than fast-growing chickens.

### Tibia Biophysical Characteristics

At 1,700 g BW class, no interaction effects between mineral source and broiler breeder strain were found on tibia biophysical characteristics (Table 7). Chickens originating from organic minerals fed broiler breeders had a higher tibia osseous volume (∆ = 1.7 cm^3^; *P* = 0.03), tibia mineral content (∆ = 1.1 g; *P* = 0.009), and tibia mineral density (∆ = 0.07 g/cm^3^; *P* = 0.003) than chickens originating from inorganic minerals fed broiler breeders. Slower growing chickens showed a higher tibia osseous volume (∆ = 7.1 cm^3^; *P* < 0.001), tibia pore volume (∆ = 1.2 cm^3^; *P <* 0.001), tibia total volume (∆ = 8.4 cm^3^; *P* < 0.001), and tibia mineral content (∆ = 1.9 g; *P* < 0.001) than fast-growing chickens.

**Table 7:** Effects of broiler breeder strain (fast-growing Ross 308 or slower growing Hubbard JA 757), dietary mineral source (inorganic or organic) and their interaction on tibia biophysical characteristics of male broiler offspring in two BW classes (1,700 and 2,600 g) (2 chickens per pen, n = 8 pens per treatment; LSmeans ± SEM).

Parameter	Tibia osseous volume (cm^3^)		Tibia pore volume (cm^3^)		Tibia total volume (cm^3^)		Tibia volume fraction (BV/TV, %)		Tibia mineral content (g)		Tibia mineral density (g/cm^3^)	
BW class	1,700 g	2,600 g	1,700 g	2,600 g	1,700 g	2,600 g	1,700 g	2,600 g	1,700 g	2,600 g	1,700 g	2,600 g
Mineral source												
Inorganic	23.0^b^	24.1	4.1	4.7	27.0	28.8	85.0	84.0^b^	11.4^b^	13.2^b^	0.24^b^	0.29
Organic	24.7^a^	27.3	4.0	4.7	28.7	32.1	86.3	85.8^a^	12.5^a^	14.6^a^	0.31^a^	0.37
SEM	0.5	0.5	0.2	0.2	0.6	0.5	0.6	0.5	0.3	0.2	0.01	0.01
Strain												
Fast	20.3^b^	21.6	3.4^b^	3.4^b^	23.7^b^	25.0	85.7	86.6^a^	11.0^b^	12.5^b^	0.26	0.31
Slower	27.4^a^	29.9	4.6^a^	6.0^a^	32.1^a^	35.9	85.6	83.2^b^	12.9^a^	15.3^a^	0.28	0.36
SEM	0.5	0.5	0.2	0.2	0.6	0.5	0.6	0.5	0.3	0.2	0.01	0.01
Mineral source × strain												
Inorganic fast	18.9	21.0^c^	3.4	3.6	22.2	24.6^c^	85.0	85.4	10.2	11.7	0.24	0.29^b^
Organic fast	21.7	22.3^c^	3.5	3.2	25.2	25.4^c^	86.4	87.7	11.8	13.4	0.28	0.32^b^
Inorganic slower	27.1	27.3^b^	4.8	5.8	31.9	33.1^b^	85.0	82.5	12.5	14.7	0.23	0.29^b^
Organic slower	27.7	32.4^a^	4.5	6.3	32.3	38.7^a^	86.1	83.9	13.2	15.9	0.34	0.42^a^
SEM	0.7	0.7	0.3	0.3	0.9	0.7	0.7	0.7	0.4	0.3	0.02	0.02
P-values												
Mineral source	0.03	<0.001	0.80	0.85	0.07	<0.001	0.08	0.03	0.009	<0.001	0.002	<0.001
Strain	<0.001	<0.001	<0.001	<0.001	<0.001	<0.001	0.84	<0.001	<0.001	<0.001	0.31	0.02
Mineral source × strain	0.12	0.01	0.51	0.10	0.15	0.005	0.83	0.59	0.25	0.33	0.13	0.03

a-c Values within a row and factor lacking a common superscript differ (*P* ≤ 0.05).

At 2,600 g BW class, interaction effects between mineral source and broiler breeder strain were found on tibia osseous volume, tibia total volume, and tibia mineral density (Table 7). In fast-growing chickens, no effect of mineral source in the breeder diet was found, but slower growing chickens originating from organic minerals fed breeders showed a higher tibia osseous volume (∆ = 5.1 cm^3^; *P* = 0.01), tibia total volume (∆ = 5.6 cm^3^; *P* = 0.005), and tibia mineral density (Δ = 0.13 g/cm3; *P* = 0.03) than chickens originating from inorganic minerals fed breeders. Chickens originating from organic minerals fed broiler breeders had a lower tibia volume fraction (∆ = 1.8 %; *P* = 0.03) and a higher tibia mineral content (∆ = 1.4 g; *P* < 0.001) than chickens originating from inorganic minerals fed broiler breeders. Slower growing chickens showed a higher tibia pore volume (∆ = 2.6 cm^3^; *P* < 0.001), tibia mineral content (∆ = 2.8 g; *P* < 0.001) and lower tibia volume fraction (∆ = 3.4 %; *P* < 0.001) than fast-growing chickens.

### Tibia Mechanical Characteristics

At 1,700 g BW class, no interaction effects between mineral source and broiler breeder strain were found on tibia mechanical characteristics (Table 8). Chickens originating from organic minerals fed broiler breeders had a higher tibia ultimate strength (∆ = 22.1 N; *P* < 0.001), tibia yield strength (∆ = 20.8 N; *P* < 0.001), tibia stiffness (∆ = 19.2 N/mm; *P* < 0.001), and tibia energy to fracture (∆ = 18.8 N-mm; *P* < 0.001) than chickens originating from inorganic minerals fed broiler breeders. Slower-growing chickens showed a higher tibia ultimate strength (∆ = 37.9 N; *P* < 0.001), tibia yield strength (∆ = 39.1 N; *P* < 0.001), tibia stiffness (∆ = 39.1 N/mm; *P* < 0.001), and tibia energy to fracture (∆ = 35.7 N-mm; *P* < 0.001) than fast-growing chickens.

**Table 8:** Effects of broiler breeder strain (fast-growing Ross 308 or slower growing Hubbard JA 757), dietary mineral source (inorganic or organic) and their interaction on tibia mechanical characteristics of male broiler offspring in two BW classes (1,700 and 2,600 g) (2 chickens per pen, n = 8 pens per treatment; LSmeans ± SEM).

Parameter	Tibia ultimate strength (N)		Tibia yield strength (N)		Tibia stiffness (N/mm)		Tibia energy to fracture (N-mm)		Tibia elastic modulus (GPa)	
BW class	1,700 g	2,600 g	1,700 g	2,600 g	1,700 g	2,600 g	1,700 g	2,600 g	1,700 g	2,600 g
Mineral source										
Inorganic	233.0^b^	266.6^b^	211.0^b^	238.9^b^	224.8^b^	237.9^b^	214.6^b^	242.6^b^	12.6	12.6
Organic	255.1^a^	280.7^a^	231.8^a^	253.7^a^	244.0^a^	253.9^a^	233.4^a^	256.8^a^	12.7	12.4
SEM	3.3	4.3	3.1	4.4	2.8	5.1	2.6	4.3	0.2	0.3
Strain										
Fast	225.1^b^	264.6^b^	201.8^b^	235.0^b^	214.9^b^	233.6^b^	206.1^b^	239.7^b^	12.8	12.8
Slower	263.0^a^	282.7^a^	240.9^a^	257.6^a^	254.0^a^	258.2^a^	241.8^a^	259.7^a^	12.5	12.1
SEM	3.3	4.3	3.1	4.4	2.8	5.1	2.6	4.3	0.2	0.3
Mineral source × strain										
Inorganic fast	216.3	257.9	194.5	226.5	207.9	224.2	199.1	233.8	12.8	12.8
Organic fast	233.9	271.4	209.2	243.5	222.0	243.1	213.2	245.5	12.8	12.9
Inorganic slower	249.6	275.4	227.4	251.2	241.8	251.7	230.0	251.3	12.4	12.4
Organic slower	276.2	290.0	254.5	263.9	266.1	264.8	253.7	268.0	12.6	11.8
SEM	4.6	6.1	4.4	6.2	3.9	7.2	3.6	6.1	0.2	0.4
*P*-values										
Mineral source	<0.001	0.04	<0.001	0.03	<0.001	0.04	<0.001	0.03	0.69	0.54
Strain	<0.001	0.008	<0.001	0.002	<0.001	0.003	<0.001	0.004	0.27	0.11
Mineral source × strain	0.35	0.93	0.18	0.74	0.21	0.70	0.20	0.69	0.60	0.39

a-b Values within a row and factor lacking a common superscript differ (*P* ≤ 0.05).

At 2,600 g BW class, no interaction effects between mineral source and broiler breeder strain were found on tibia mechanical characteristics (Table 8). Chickens originating from organic minerals fed broiler breeders had a higher tibia ultimate strength (∆ = 14.1 N; *P* = 0.04), tibia yield strength (∆ = 14.8 N; *P* = 0.03), tibia stiffness (∆ = 16.0 N/mm; *P* = 0.04), and tibia energy to fracture (∆ = 14.2 N-mm; *P* = 0.03) than chickens originating from inorganic minerals fed broiler breeders. Slower growing chickens showed a higher tibia ultimate strength (∆ = 18.1 N; *P* = 0.008), tibia yield strength (∆ = 22.6 N; *P* < 0.002), tibia stiffness (∆ = 24.6 N/mm; *P* = 0.003), and tibia energy to fracture (∆ = 20.0 N-mm; *P* = 0.004) than fast-growing chickens.

### Home Pen Behavior

Home pen behavior parameters (eating, drinking, walking, standing, resting, sitting, dust bathing, and perching) for each of the observation days (d 16, 23, 30, 37, and 44) are presented in Table A2 in the Appendix. Hardly any effect of mineral source in the breeder diet was found on behavior parameters. Fast-growing chickens showed less walking (d 30 and 37), less standing (all days), less perching (all days), and more resting behavior (d 30) than slower growing chickens.

### Leg Disorders

No interaction effects between mineral source and broiler breeder strain were found on VV and TD at both 1,700 and 2,600 g BW classes and furthermore, no main effects were found on TD. At 1,700 g and 2,600 g BW classes, fast-growing chickens showed a higher VV incidence than slower-growing chickens (1,700 g: 25.0 vs. 4.2%, respectively; *P* = 0.02; 2,600 g: 37.5 vs. 23.0%, respectively; *P* = 0.04).

## DISCUSSION

### Minerals in Eggs and Hatchlings

Results of mineral analyses in the current study demonstrated that source of the macro and trace minerals in the broiler breeder diet affected the concentrations of some minerals in eggs of both fast and slower growing broiler breeders. Fe and Se concentrations were found higher in the mixture of egg content, whereas Cu was found lower in breeders fed organic minimum compared to those fed inorganic mineral source. The other minerals (Ca, P, Mn, Zn) were not influenced by mineral source in the breeder diet nor by broiler breeder strain. The yolk is the main source of nutrients for the embryo and supplies phosphorus and trace minerals, while the eggshell is the main calcium source (Torres and Korver, 2018). Studies have shown that increasing the concentration of macro and trace minerals in the diet of broiler breeder or laying hens hardly influenced their concentrations in the egg, since the amounts of minerals in the egg have certain limits, which is mainly determined by the genetic background of the breeder (Naber, 1979; Angel, 2007).

Changing the mineral source in the maternal diet might be an alternative way to influence mineral concentrations in the egg and consequently in the offspring. Effects of trace mineral source in broiler breeder diets on mineral concentrations in their eggs are hardly investigated, whereas studies on macro mineral source in broiler breeder diets are completely lacking. Furthermore, the exact pathways of macro and trace mineral transfer from the hen to the egg yolk, albumen, and egg shell are still unclear (Dacke et al., 2015) and even more interesting, it is unclear whether or not pathways of inorganic and organic mineral transfer from breeder to egg differ. Results of the current study about iron and selenium concentration in the egg are in line with previous studies, indicating that organic selenium was found to be transferred more efficiently from broiler breeders (Pappas et al., 2005) and laying hens (Sirichakwal et al., 1984; Miles, 2001; Kidd, 2003) to the egg than inorganic selenium. Organic iron in laying hen diet resulted in higher amount of iron in eggs (Park et al., 2004; Buckiuniene et al., 2017) than inorganic iron in the laying diet. These and our findings suggest that organic selenium and iron in the broiler breeder diet affect the physiological transfer and deposition pathways and finally ends up with increased concentrations in the egg. Regarding manganese, copper, and zinc, several studies with laying hens have shown that an organic form of those minerals in the diet resulted in higher concentrations in eggs than an inorganic form (Dobrzanski et al., 2008; Venglovska et al., 2014; Yenice et al., 2015; Saleh et al., 2019), but this increase was not found in the current study. This may be explained by different genetic backgrounds between broiler breeders and laying hens, but further investigation is needed to understand the exact pathways.

Looking at the results of residual yolk and yolk free bodies of hatchlings, selenium appeared to be the only mineral which was affected by the mineral source in the breeder diet in both residual yolk and yolk free bodies. It can be speculated that the higher concentration of selenium in eggs was effectively absorbed and retained in the yolk free body during embryonic development and also more selenium was still left in the residual yolk. The fact that only selenium was affected in the hatchling body and not the other minerals might indicate that selective absorption of minerals through the yolk sac membrane into the blood stream is occurring (Bokkers and Koene, 2003; Yair and Uni, 2013). However, the exact pathways and factors affecting these pathways remain unclear and need to be investigated.

Despite the fact that breeder strain did not show any effect on mineral concentration in the mixture of yolk and albumen nor the eggshell, differences in mineral concentration were found in the residual yolk and yolk free body, but effects were ambiguous. Calcium, copper, and manganese were higher in residual yolk of slower growing hatchlings compared to fast-growing hatchlings, whereas the opposite was found for phosphorus, iron and selenium. This again suggests that selective absorption of minerals takes place as indicated above.

Based on the mineral concentrations in the egg, residual yolk, and yolk free body, it can be concluded that effects of mineral sources in the breeders’ diet on concentrations in the eggs and hatchlings are marginal. However, effects on BW and tibia characteristics are found (see below), suggesting that other mechanisms than only mineral concentration appears to play a role in trans-generational mineral transfer in broiler chickens.

### Growth Performance

Although effects of mineral source in the broiler breeder diet were limited in relation to mineral concentrations in both eggs and hatchlings, effects on offspring BW were evident, particularly in the slower growing chickens. From d 10 of age onward, in the slower growing strain, chickens originating from organic minerals fed broiler breeders had higher BW than chickens originating from inorganic minerals fed broiler breeders, while this effect was not seen in fast-growing chickens. Earlier studies demonstrated that organic trace minerals had a higher bioavailability in fast-growing breeders and finally resulted in higher post-hatch growth performance (Chang et al., 2016; M'Sadeq et al., 2018; Araújo et al., 2019) than inorganic trace minerals. It can be speculated why offspring growth rate in the current study was positively affected by organic minerals in the breeder diet of the only slower growing strain and not in the fast-growing strain. The first reason, might be related to the difference in feed intake, and thus in mineral intake, between fast and slower growing broiler breeders. It can be speculated that with a lower feed intake in the slower growing breeders, the mineral intake was too low in the inorganic diet and with the organic diet, with a higher mineral availability, this lack of sufficient mineral intake has been reduced. Second, mineral absorption, deposition or mineral transferring physiological pathways between slower and fast-growing chickens differ, because of their different genetic backgrounds (Yair et al., 2013; Torres and Korver, 2018). Third, it might be that slower growing chickens with better developed bones are more active and better able to reach the feed and water. This is supported by the numerically higher FI in the slower growing chickens originating from organic minerals fed breeders compared to the ones originating from inorganic minerals fed breeders. It appears that organic minerals in broiler breeder diets and sufficiently developed and mineralised bones could work together to reach a better growth performance.

Regarding the strain, in the current study, fast-growing broiler chickens, as expected, showed higher BW and feed efficiency compared to the slower growing broiler chickens on the same ages. This is in accordance with previous studies showing that fast-growing chickens have been specifically selected for these 2 parameters (Bokkers and Koene, 2003; Quentin et al., 2004; Benyi et al., 2010, 2015).

### Tibia Characteristics

Results of the current study provided evidence that organic macro and trace minerals in the maternal diet of slower growing chickens resulted in better offspring tibia characteristics compared to inorganic form of those minerals in the maternal diet. These findings are in line with previous research, indicating that a higher trace mineral availability in the diet of broiler breeder leads to more advanced bone development of their offspring from embryonic phase till slaughter age (Sirichakwal et al., 1984; Dibner et al., 2007; Torres and Korver, 2018; Saleh et al., 2019). Changing the mineral sources from inorganic to organic has been found to positively affect embryonic bone development and later life leg health, due to their higher mineral mobilization and bioavailability (Kidd et al., 1992; Park et al., 2004; Echigo and Kimata, 2010; Favero et al., 2013; Oviedo-Rondón et al., 2013). The main reason of the higher mobilization and bioavailability of organic minerals compared to inorganic minerals is related to their bonds. Organic minerals contain covalent bonds that provide a better binding strength with other compounds and result in better chemical stability compared to inorganic minerals, which are bound by weak electrovalent and/or ionic bonds (Vieira, 2008; Bao and Choct, 2009; Echigo and Kimata, 2010; Wang et al., 2019). Despite the fact that concentrations of minerals in the eggs and hatchlings originated from organic and inorganic minerals fed broiler breeders hardly differed, it appears that other mechanisms have played a role on post-hatch BW gain and bone development, but these mechanisms are currently unclear.

Regarding the strain, slower growing chickens showed better tibia morphological, biophysical, and mechanical characteristics compared to fast-growing chickens at both 1,700 and 2,600 g BW classes. This difference can probably be explained by the negative correlation between growth rate and bone development. Fast-growing broiler chickens have been shown to have poorer mineralized bones compared to slower growing broilers (Lilburn, 1994; Velleman, 2000; Bonser and Casinos, 2003). Fast growth is known to result in poorer mineralized bones, due to the fact that mechanisms involved in bone development cannot keep up with fast growth of the broiler, particularly during the first 2 wk of the growth phase. Slower growth ensures that there is more time for bone mineralization, which compensates for the lack of mineralization in the early growth phase (Shim et al., 2012b; Sanchez-Rodriguez et al., 2019).

In conclusion, despite the fact that effects on mineral concentration in eggs and hatchlings were limited, organic macro and trace minerals in the broiler breeder diet showed positive effects on both offspring BW and tibia characteristics in slower growing chickens, whereas this effect was hardly seen in fast-growing chickens. This suggests that 1) the difference in feed intake between fast and slower growing broiler breeders might affect offspring performance, which might indicate that current slower growing broiler breeder diets might be suboptimal in minerals or that transgenerational mineral availability in slower growing chickens appears to be more effective on bone development than in fast-growing chickens, which might be related to time available for bone development. 2) transgenerational mineral availability in offspring appears to play a role via other mechanisms than via absolute mineral concentrations in the egg.

## Appendix Supplementary materials

Image, application 1
