# Supplementary material for: Effects of organic macro and trace minerals in fast and slower growing broiler breeders’ diet on offspring growth performance and tibia characteristics
Source: Poult Sci. 2021 Dec 9;101(3):101647. doi: 10.1016/j.psj.2021.101647 (PMC8749331; doi:10.1016/j.psj.2021.101647)
Supplement: Supplementary file 1 [file mmc1.docx]

**Appendix**

Table A1. Effects of broiler breeder strain (fast-growing Ross 308 or slower-growing Hubbard JA 757), dietary mineral source (inorganic or organic) and their interaction on hatchling characteristics (LSmeans±SEM).

| Parameter | n | Egg weight^1^  (g) | Red hock^2^ (%) | Red beak^2^ (%) | Navel score^3^ |
| --- | --- | --- | --- | --- | --- |
| Mineral source |  |  |  |  |  |
| Inorganic | 517 | 53.5^a^ | 5.06 | 6.19 | 1.21 |
| Organic | 515 | 52.7^b^ | 6.62 | 6.80 | 1.20 |
| SEM | - | 0.22 | 1.03 | 1.10 | 0.02 |
| Strain |  |  |  |  |  |
| Fast | 498 | 55.5^a^ | 3.97^b^ | 4.97^b^ | 1.19 |
| Slower | 534 | 50.6^b^ | 7.70^a^ | 8.03^a^ | 1.22 |
| SEM | - | 0.22 | 1.03 | 1.10 | 0.02 |
| Mineral source*strain |  |  |  |  |  |
| Inorganic fast | 238 | 56.1 | 2.94 | 3.78 | 1.19 |
| Organic fast | 259 | 55.0 | 5.00 | 6.15 | 1.20 |
| Inorganic slower | 278 | 50.9 | 7.17 | 8.60 | 1.23 |
| Organic slower | 257 | 50.4 | 8.24 | 7.45 | 1.21 |
| SEM | - | 0.31 | 1.47 | 1.53 | 0.03 |
| P-values |  |  |  |  |  |
| Mineral source | - | 0.03 | 0.29 | 0.70 | 0.83 |
| Strain | - | <0.001 | 0.02 | 0.05 | 0.35 |
| Mineral source*strain | - | 0.44 | 0.74 | 0.26 | 0.74 |

^a-b^ Values within a column and factor, lacking a common superscript differ (*P*≤0.05).

^1^ Average egg weight per treatment before incubation by weighing egg trays.

^2^ Red hock and red beak of all hatchlings were scored as 0 (absent) or 1 (present) and expressed as percentage of chickens with red hocks or red beaks.

^3^ Navel score was assessed as 1 (good), 2 (moderate) or 3 (poor), analysed binary (good vs moderate plus poor) and expressed as LSmeans.

Table A2. Effects of broiler breeder strain (fast-growing Ross 308 or slower-growing Hubbard JA 757) and dietary mineral source (inorganic or organic) on percentage of male offspring chickens showing the following behaviours in their home pen (eating, drinking, walking, standing, resting, sitting, dustbathing and perching) at day 16, 23, 30, 37 and 44 of age (all chickens in the pen, n=8 pens per treatment)

| Parameter and day | Mineral source | | Strain | | SEM | *P*-values^2^ | |
| --- | --- | --- | --- | --- | --- | --- | --- |
|  | Inorganic | Organic | Fast | Slower |  | Mineral source | Strain |
| Eating |  |  |  |  |  |  |  |
| 16 d | 3.46 | 2.60 | 4.17 | 1.90 | 1.13 | 0.60 | 0.17 |
| 23 d | 2.65 | 2.33 | 4.47^a^ | 0.51^b^ | 0.83 | 0.79 | 0.003 |
| 30 d | 5.70 | 3.06 | 6.57 | 2.19 | 1.59 | 0.25 | 0.06 |
| 37 d | 4.84 | 3.83 | 6.56 | 2.11 | 1.64 | 0.67 | 0.06 |
| 44 d^1^ | 2.78 | 0.00 | - | - | 1.52 | 0.21 | - |
| Drinking |  |  |  |  |  |  |  |
| 16 d | 4.50 | 2.86 | 4.69 | 2.68 | 1.32 | 0.39 | 0.29 |
| 23 d | 4.05 | 7.22 | 6.84 | 4.42 | 1.35 | 0.11 | 0.22 |
| 30 d | 3.59 | 2.42 | 2.78 | 3.23 | 1.20 | 0.50 | 0.79 |
| 37 d | 3.58 | 3.41 | 4.25 | 2.75 | 1.50 | 0.94 | 0.49 |
| 44 d^1^ | 0.70 | 0.00 | - | - | 0.49 | 0.33 | - |
| Walking |  |  |  |  |  |  |  |
| 16 d | 7.60 | 7.77 | 6.25 | 9.12 | 1.73 | 0.95 | 0.25 |
| 23 d | 8.74 | 10.81 | 8.40 | 11.14 | 2.02 | 0.48 | 0.35 |
| 30 d | 6.22 | 7.58 | 3.86^b^ | 9.94^a^ | 1.92 | 0.62 | 0.03 |
| 37 d | 4.42 | 2.40 | 0.35^b^ | 6.47^a^ | 1.07 | 0.19 | <0.001 |
| 44 d^1^ | 8.12 | 6.61 | - | - | 3.10 | 0.74 | - |
| Standing |  |  |  |  |  |  |  |
| 16 d | 9.50 | 7.29 | 3.91^b^ | 12.88^a^ | 1.77 | 0.39 | 0.002 |
| 23 d | 3.70 | 7.46 | 3.41^b^ | 7.75^a^ | 1.50 | 0.09 | 0.05 |
| 30 d | 3.80 | 5.51 | 1.82^b^ | 7.49^a^ | 1.69 | 0.48 | 0.03 |
| 37 d | 5.27 | 3.68 | 2.47^b^ | 6.47^a^ | 1.31 | 0.40 | 0.04 |
| 44 d^1^ | 2.37 | 5.48 | - | - | 1.83 | 0.24 | - |
| Resting |  |  |  |  |  |  |  |
| 16 d | 12.98 | 10.16 | 13.92 | 9.21 | 1.99 | 0.33 | 0.11 |
| 23 d | 18.82^a^ | 9.00^b^ | 14.42 | 13.40 | 2.62 | 0.02 | 0.79 |
| 30 d | 11.75 | 15.29 | 19.36^a^ | 7.67^b^ | 2.69 | 0.36 | 0.004 |
| 37 d | 9.08 | 10.56 | 11.33 | 8.31 | 2.53 | 0.68 | 0.41 |
| 44 d^1^ | 7.81 | 8.86 | - | - | 3.65 | 0.85 | - |
| Sitting |  |  |  |  |  |  |  |
| 16 d | 57.19 | 63.20 | 63.79 | 56.60 | 3.41 | 0.23 | 0.15 |
| 23 d | 55.45 | 58.24 | 55.45 | 55.24 | 3.56 | 0.59 | 0.52 |
| 30 d | 64.73 | 62.96 | 65.61 | 62.08 | 3.26 | 0.71 | 0.45 |
| 37 d | 69.54 | 73.33 | 74.00 | 68.88 | 3.46 | 0.30 | 0.45 |
| 44 d^1^ | 75.97 | 77.57 | - | - | 5.3 | 0.84 | - |
| Dust bathing |  |  |  |  |  |  |  |
| 16 d | 1.59 | 2.49 | 2.75 | 1.33 | 1.30 | 0.63 | 0.45 |
| 23 d | 2.37 | 2.60 | 2.89 | 2.08 | 0.81 | 0.84 | 0.49 |
| 30 d | 0.26 | 0.26 | 0.00 | 0.52 | 0.26 | 1.00 | 0.16 |
| 37 d | 1.39 | 0.69 | 1.04 | 1.04 | 0.85 | 0.57 | 1.00 |
| 44 d^1^ | 0.69 | 0.69 | - | - | 0.69 | 1.00 | - |
| Perching |  |  |  |  |  |  |  |
| 16 d | 3.20 | 3.62 | 0.52^b^ | 6.30^a^ | 1.40 | 0.84 | 0.007 |
| 23 d | 4.24 | 2.34 | 1.07 | 5.51 | 1.56 | 0.40 | 0.06 |
| 30 d | 3.97 | 2.92 | 0.00^b^ | 6.88^a^ | 1.18 | 0.54 | <0.001 |
| 37 d | 1.89 | 2.08 | 0.00^b^ | 3.98^a^ | 0.90 | 0.89 | 0.003 |
| 44 d^1^ | 1.56 | 0.78 | - | - | 1.24 | 0.66 | - |

^a-b^ Values within a row and factor, lacking a common superscript differ (*P*≤0.05).

^1^ Only slower-growing chickens.

^2^ No interactions between broiler breeder strain and mineral source were observed for any of the behaviours and any of the sampling days.
